# A randomized phase II study of radiation induced immune boost in operable non-small cell lung cancer (RadImmune trial)

**DOI:** 10.1186/s12885-015-2006-2

**Published:** 2015-12-19

**Authors:** Seyer Safi, Philipp Beckhove, Arne Warth, Axel Benner, Falk Roeder, Stefan Rieken, Juergen Debus, Hendrik Dienemann, Hans Hoffmann, Peter E. Huber

**Affiliations:** 1 Department of Thoracic Surgery, Thoraxklinik, Heidelberg University Hospital, Heidelberg, Germany; 2 Translational Immunology Unit, German Cancer Research Center, Heidelberg, Germany; 3 Division of Thoracic Pathology, Institute of Pathology, Heidelberg, Germany; 4 Division of Biostatistics, German Cancer Research Center, Heidelberg, Germany; 5 Departments of Molecular and Radiation Oncology, Heidelberg University Hospital and German Cancer Research Center, Heidelberg, Germany; 6 Present address: Department Radiation Oncology, University Hospital Munich (LMU), Munich, Germany

**Keywords:** Lung cancer, Immunotherapy, Low dose radiation, T cells

## Abstract

**Background:**

Lung cancer is the leading cause of cancer deaths worldwide. Surgery, radiotherapy at conventional and high dose and chemotherapy are the mainstay for lung cancer treatment. Insufficient migration and activation of tumour specific effector T cells seem to be important reasons for inadequate host anti-tumour immune response. Ionizing radiation can induce a variety of immune responses. The goal of this randomized trial is to assess if a preoperative single fraction low dose radiation is able to improve anti-tumour immune response in operable early stage lung cancer.

**Methods/Design:**

This trial has been designed as an investigator-initiated, prospective, randomized, 2-armed phase II trial. Patients who are candidates for elective resection of early stage non-small cell lung cancer will be randomized into 2 arms. A total of 36 patients will be enrolled. The patients receive either 2 Gy or no radiation prescribed to their primary tumour. Radiation will be delivered by external beam radiotherapy using 3D radiotherapy or intensity-modulated radiation technique (IMRT) 7 days prior to surgical resection. The primary objective is to compare CD8+ T cell counts detected by immunohistochemistry in resected tumours following preoperative radiotherapy versus no radiotherapy. Secondary objectives include the association between CD8+ T cell counts and progression free survival, the correlation of CD8+ T cell counts quantified by immunohistochemistry and flow cytometry, local tumour control and recurrence patterns, survival, radiogenic treatment toxicity and postoperative morbidity and mortality. Further, frequencies of tumour reactive T cells in blood and bone marrow as well as whole blood cell transcriptomics and plasma-proteomics will be correlated with clinical outcome.

**Discussion:**

This unique intervention combining preoperative low dose radiation and surgical removal of early stage non-small cell lung cancer is designed to address the problem of inadequate host anti-tumour immune response. If successful, this study may affect the role of radiotherapy in lung cancer treatment.

**Trial registration:**

NCT02319408; Registration: December 29, 2014.

## Background

Lung cancer causes more deaths than colon, breast and prostate cancer together [1]. Non-small cell lung cancer is the most common type of lung cancer and accounts for approximately 81 % of all cases of lung cancer [2].

Close to 58 % of patients with NSCLC present with locally advanced or metastatic disease (stages IIIB and IV) at the time of diagnosis [3]. By then, the disease is generally incurable. Even in early stages of disease, the median time to tumour relapse after complete surgical resection is two years. The 5-year survival rate is 73 % in stage IA, 58 % in stage IB and 46 % in stage IIA. However, this rate drops to 36 % in stage IIB and 24 % in stage IIIA [4].

Currently four cycles of adjuvant platin-based chemotherapy is recommended in patients with completely resected stage II-IIIA NSCLC [5]. However, its administration only provides a 5-year absolute survival benefit of 4 % [6]. Moreover, due to medical comorbidities, age, poor performance status or postoperative complications a significant amount of patients is not eligible for adjuvant chemotherapy [7].

The 5-year recurrence-free rate even in stage I NSCLC is only about 75 % [8]. Therefore the development of postoperative treatments represents a so far unmet medical need for patients with resected stage I-IIA NSCLC as well as for patients with stage IIB-IIIA NSCLC, which will not be included into this study.

Radiotherapy (RT) is an important treatment modality for localized tumours. RT typically induces primarily mitotic cell death but also leads to apoptosis [9] and complex effects on the tumour microenvironment, which can facilitate homing of both antigen presenting cells and effector T cells [10]. Sublethal doses of ionizing radiation have been described to directly stimulate major histocompatibility complex (MHC) expression that renders tumour cells more sensitive to detection and lysis by specific T cells [11].

Tumour antigen specific cytotoxic and T helper cells are spontaneously generated in many cancer patients and provide the basis for T cell accumulation in tumour tissue [12–22]. Increased tumour infiltration by endogenous T cells has been associated with prognosis in several solid cancer types [23]. In NSCLC the presence of tumour infiltrating lymphocytes has been shown to be associated with improved outcome [24–32]. However, T cell based immunotherapies are limited by insufficient frequencies of tumour infiltrating T cells in NSCLC [33].

RT may provide a local anti-tumour immune effect and modulate the tumour microenvironment. Furthermore, preclinical work in murine models suggests that local radiotherapy plus intratumoural syngeneic dendritic cells (DC) injection can improve immunologic tumour eradication [34].

In two other tumour entities, namely in resectable liver metastases from colorectal cancer [35] and resectable pancreatic cancer [36] analogue studies are recruiting with promising preliminary results [37].

In this trial the impact of preoperative low dose radiation on immune response will be tested in lung cancer patients with cN0 lymph nodes. Preoperative radiation is commonly given before surgery for stage III lung cancer as per the intergroup trial 0139. The latter randomized controlled trial was conducted to test the value of surgery after preoperative concurrent chemotherapy/45 Gy radiotherapy for stage III N2 lung cancer. Patients who underwent radical lobectomy compared to those who received completion radiation up to 61 Gy showed a better long term overall survival [38]. However, whether chemotherapy, radiation, surgery or a combination of these treatments and which biological mechanism contributed to the survival benefit remains unclear. Our trial will focus on the biological phenomena that may contribute to overcoming the immunosuppressive processes that prevent an effective anti-tumour response.

While high radiation doses, ~20 Gy and above single dose, are thought to induce immune response via necrosis or other mechanisms, low dose radiation, more than 0.5 Gy and less than 5 Gy, may evoke immune response by polarization change of macrophages and subsequent T cell invasion. Data from our earlier studies suggest that doses around 2 Gy are sufficient to provoke a marked T cell invasion in irradiated tumours [35, 36, 39]. Furthermore 2 Gy is the standard single fraction dose in our clinical setting. So far, no systematic clinical analysis has been performed to investigate the immune stimulatory effect of low dose radiotherapy in patients with NSCLC. Therefore, for both biological and practical reasons, we suggest here to test the hypothesis that a single 2 Gy fraction of external beam radiation administered 1 week prior to lobectomy will improve anti-tumour immune responses in patients with resectable early stage non-small cell lung cancer.

## Methods/design

### Patient selection criteria

This single institutional trial focuses on patients with operable early stage NSCLC. Patients scheduled for elective lobectomy at Thoraxklinik Heidelberg, a specialized lung cancer hospital at Heidelberg Comprehensive Cancer Center, may be enrolled in this study if all inclusion criteria are fulfilled.

Potential immune-mediated diseases are exclusion criteria. They include autoimmune diseases and other inflammatory and neurologic disorders that may have an autoimmune aetiology [40]. The patient must be excluded if any exclusion criterion applies.

### Inclusion criteria

- ♦Histologically proven clinical stage I to IIA pulmonary adenocarcinoma
- ♦Lung tumour is felt to be curatively resectable by the treating physicians
- ♦Sufficient pulmonary function for lobectomy according to current guidelines [41]
- ♦The patient is free of distant metastases as confirmed by contrast-enhanced chest and upper abdomen CT-scan and by contrast-enhanced CT or MRI of the brain
- ♦Age 40 at the time of consent due to federal radiation protection law
- ♦In female patients of childbearing potential there must be a negative pregnancy test
- ♦ECOG performance status of 0,1, 2 or 3 at the time of randomization
- ♦Patients who the investigator believes can and will comply with the requirements of this protocol
- ♦Written informed consent according to ICH/GCP and national/regional regulations

### Exclusion criteria

- ♦The patient shows clinical signs of pneumonia
- ♦The patient receives immunosuppressive drugs (alkylating agents, antimetabolites, methotrexate, azathioprine, mercaptopurine, cytotoxic antibodies, ciclosporin, tacrolimus, sirolimus, interferon, mycophenolate, small biological agents)
- ♦The patient receives oral or inhaled steroids on a regular basis
- ♦The patient has been diagnosed with a potential immune mediated disease
- ♦Elevated blood leukocyte count or erythrocyte sedimentation rate [42]
- ♦Pregnancy
- ♦The patient has received any cancer specific treatment, including radiotherapy, thermal ablation, hyperthermia, immunotherapy, hormonal therapy or chemotherapy
- ♦The patient is diagnosed with a concomitant malignancy and/or has a history of malignancy within the past five years or has had a malignancy that has been in complete remission for less than 5 years
- ♦The patient needs chronic long term oxygen therapy
- ♦The patient has undergone splenectomy
- ♦The patient is known to be HIV positive
- ♦The patient has an uncontrolled bleeding disorder
- ♦The patient received any anti-infectious vaccination in the last 6 months

### Trial design

**Fig. 1:**
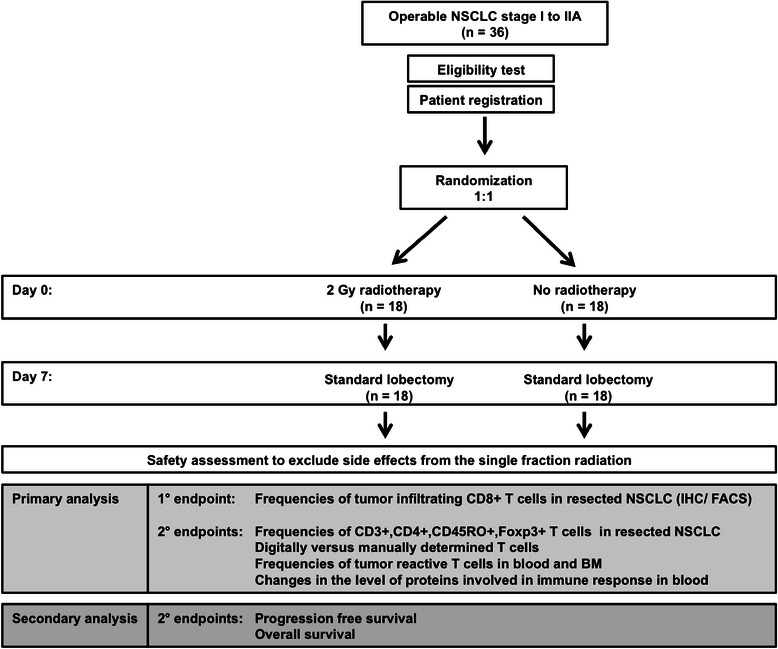
Enrolment, randomization, treatment of study participants and analysis

This is a randomized phase II single centre trial comparing the frequencies of tumour infiltrating CD8+ T cells in early stage NSCLC patients receiving 2 Gy preoperative radiotherapy versus no preoperative radiotherapy. The randomization ratio is 1:1 (Fig. 1).

### Procedures

#### Radiotherapy

Within the radiotherapy treatment arm patients will receive a single fraction of radiation on day 0 ± 2 days prescribed to their primary tumours only which are going to be removed later in the surgical procedure. The single fraction radiation will be administered 7 ± 2 days prior to operative tumour resection. After the adjustment of an individual positioning device, computed tomography and, if needed, magnetic resonance imaging (MRI) treatment planning examinations are performed. After treatment planning 3D radiotherapy or IMRT will be administered using 6-megavolt linear acceleration (LINAC) photon beams.

#### Safety aspects and adverse events

Adverse events (AEs) rarely occur in conventional radiotherapy using considerably higher doses (e.g. 30 x 2 Gy and higher versus 2 Gy in this trial) [43]. Similarly, a single dose of more than 30 Gy is prescribed for small lung tumours as definitive treatment in patients who are no candidates for surgery with an equally low side effect profile. Potential AEs usually occurring in conventional radiotherapy at higher thresholds include acute and subacute skin alterations like redness, temporarily coughing, transient nausea or diarrhoea, and pneumonitis. Medication for symptomatic treatment, e.g. antitussive drugs, is permitted. Chronic side effects upon conventional high dose radiotherapy including lung fibrosis can be excluded here given the low dose single fraction and healthy tissue sparing technique. Thus no relevant toxicities are expected.

#### Surgery

The operation is not a study procedure. Lobectomy with systematic lymph node dissection is considered the most effective treatment for early stage NSCLC [5]. After low dose radiotherapy on day 0 ± 2 days, surgery will be performed on day 7 ± 2 days. In brief, patients undergo general anaesthesia and endobronchial double-lumen intubation. Blood pressure, heart rate, electrocardiographic trace, respiratory frequency and oxygen saturation are monitored continuously throughout the operation by an anaesthesiologist. The entire lung consists of two lobes on the left and three lobes on the right side. Thoracotomy refers to surgical opening of the chest usually through the fourth or fifth intercostal space. Vascular and bronchial structures at the level of the hilum are prepared and resected along with the corresponding lobe and lymph node containing fatty tissues. A radical systematic lymph node dissection accomplishes a standard lobectomy procedure. In special cases video-assisted thoracic surgery (minimal-invasive; small incisions) may replace open thoracotomy (larger incision). However, there is no difference between the methods in terms of tumour manipulation or even tumour cell spread.

Accurate assessment of lymph node status is a key-factor for staging of NSCLC and its surgical outcome. Therefore in this study all patients undergoing standard lobectomy via anterolateral thoracotomy or video-assisted thoracoscopic surgery will receive radical mediastinal and hilar lymphadenectomy [44] .

### Timeline

#### Patient screening

Only patients with stage I-IIA NSCLC, who, in the opinion of the investigators, are likely to meet all inclusion and none of the exclusion criteria may be included in this study on day −30 to day 0. All patients will be staged with PET/CT. Lymph nodes with pathological findings are routinely examined via bronchoscopy and fine needle aspiration or mediastinoscopy. Only patients that are clinically N0 will be enrolled. Patients should receive detailed information about the study and the study procedures and should be proposed to sign the declaration of informed consent to participate in the full study.

#### Registration, randomization and single fraction radiotherapy

Patients will be registered on study and equally randomized to the two treatment arms. Randomization will be done on day −14 ± 2 days. Patients in the preoperative radiation group will be seen on day −7 ± 2 days at the radiotherapy department including radiation treatment planning and receive a single fraction radiation on day 0 ± 2 days.

#### Preoperative assessment

Each patient will be admitted to hospital on day 6 ± 2 days (one day before surgery) and seen by an anaesthesiologist. For study purposes bone marrow blood will be taken under sterile conditions and local anaesthesia.

#### Surgery

Each patient will undergo standard lobectomy with radical lymph node dissection on day 7 ± 2 days.

#### Postoperative visit

A patient will be considered to have completed the treatment when he/she is deemed stable and has reached the end of all scheduled study visits and the follow-up contact. If no postoperative complications occur this may be day 21 ± 7 days. Primary analysis of data and report of these results will be conducted after the time when the last patient completed the day 21 visit.

#### Long-term follow up

After the first postoperative visit patients will be seen on an outpatient clinic basis every 3 months for 2 years and then every 6 months for a total of 6 years. A secondary analysis of long-term follow-up data will be conducted to report on PFS and OS. The end of the entire study is defined as when patient follow-up ends with last patient completing study. The clinical trial is ended when data analysis is completed and reported.

#### Ethical and legal considerations

The independent ethics committee of the University of Heidelberg approved the final clinical trial protocol, the patient information and informed consent sheets. Written informed consent is obtained from each patient in oral and written form before inclusion in the study. Patients are informed about the strict confidentiality of their personal data within this trial, but their pseudonymised medical records may be reviewed for trial purposes by authorized individuals other than their treating physician.

Participation to this trial is voluntary and patients are allowed to refuse further participation at any time point within the study. This trial is carried out in accordance to the current Declaration of Helsinki (sixth revision, 2008), the principles of "Good Clinical Practice" (GCP), and the Federal Data Protection Act. This study follows the CONSORT guidelines and is registered at the Clinical-Trials.gov protocol registration system (identification number: NCT02319408).

### Evaluation criteria/endpoints

Tumour infiltrating lymphocytes will be investigated as described by Halama et al. [45]. Tissue specimens will be immunohistochemically analysed for their infiltration of CD8+ (primary endpoint), CD3+, CD4+, CD45RO+ and Foxp3+ T cells (secondary endpoints) in tissue sections from formalin-fixed, paraffin-embedded tissue.

### Analysis of immunohistochemistry data

The number of stained immune cells will be counted using a computerized image analysis system attached to a personal computer. Complete microscopic images of full tissue sections will be automatically obtained for later automatic or manual visual analysis (virtual microscopy), allowing large-scale histologic evaluation with high precision across the complete section. Thus, varying cell densities across the complete tissue section can be measured objectively. In this analysis, the average cell density across the measured region will be used for analysis. Manual evaluation of stained immune cells will be conducted in duplicates without knowledge of the clinicopathological data pathologist-blinded. Manual cell counts will be reassessed with a specifically developed software program to measure cell densities across a given region of interest. This will be done to confirm the manual cell counts, so that the automated quantification application can be validated.

### Other secondary endpoints

Other secondary endpoints are digitally versus manually determined CD8+ T cell frequencies in resected NSCLC tumours, frequencies of tumour reactive T cells in blood and bone marrow before radiotherapy and after surgery, changes in the level of proteins involved in immune response in blood determined by ELISA and frequencies of CD3+, CD4+, CD8+, CD45RO and Foxp3+ T cells in resected NSCLC tumours determined by flow cytometry. Depending on the availability of pre-enrolment harvested tumour tissue, the immune contexture of these tumour biopsies will be compared to the post-radiation samples obtained from surgery. Secondary analysis will be conducted for progression free survival and overall survival as secondary endpoints, which require long-term follow-up of 6 years and will be performed and reported subsequent to the primary analysis, as well as other laboratory data derived from proteomic or transcriptomic analyses. Whole blood transcriptomics will be performed as described earlier [46]. Peripheral blood samples will be obtained before radiation, at the time of surgery, and the first visit of the patient at the radiation oncology department, the total RNA will be extracted from whole blood and the resulting expression profiles will be correlated with the immune response and clinical status of the patients. As for the proteome an array of 20 cytokines with known roles in immune response will be measured using a commercial protein array at the same three time points. Tumour reactive T cells will be determined in bone marrow aspirates by ELIPOT analysis. Bone marrow aspirates will be taken one to three days before radiation and under anaesthesia before skin incision.

### Study design

This is a two-arm randomized phase II study to investigate the influence of low dose radiotherapy on immune stimulatory effects in NSCLC. The protocol will accrue up to 36 response analysable patients. Patients will be randomized to one of the following groups: 1. Control arm: no preoperative radiotherapy and 2. Experimental arm: 2 Gy single fraction preoperative radiotherapy administered to patients 7 days prior to surgery. The primary objective is to assess the effect of local low dose radiotherapy on tumour infiltrating CD8+ T cells (cells/mm^2^) as a surrogate measure for anti-tumour activity.

### Randomization and stratifications

A block randomization will be conducted by computer randomization. There is no stratification for randomization. The randomization ratio is 1:1.

### Sample size determination

Based on information of the study by Galon et al. [47] in colorectal adenocarcinoma the presence of tumour infiltrating T cells is associated with improved patient outcome. Patients with a good prognosis had a mean of 600 CD8+ T cells/mm^2^ and patients with a bad prognosis had a mean of 370 CD 8+ T cells/mm^2^. The standard deviation in both groups was 50 cells/mm^2^. Their analysis further revealed a cut-off point: Patients with a frequency of 300 tumour infiltrating CD8+ T-cells/mm^2^ had a significantly better prognosis. The difference between prognostic groups and identified threshold are not assumed to translate directly to giving 2 Gy single fraction preoperative radiotherapy in NSCLC, but the standard deviation is used for sample size estimation in the actual study.

The null hypothesis (H0) is that in patients with operable NSCLC a single fraction low dose preoperative radiotherapy does not increase the frequency of tumour infiltrating CD8+ T cells. The alternative hypothesis (Ha) used for power computation is that in patients with operable NSCLC a single fraction low dose preoperative radiotherapy increases the frequency of tumour infiltrating CD8+ T cells at least by 50 CD8+ T cells/mm^2^. For both treatment arms we assume a standard deviation of 50 cells/mm^2^.

Given that the purpose of this study is to determine whether preoperative radiotherapy should be studied further, there is only one outcome of interest, superiority of the experimental arm to the control. In this scenario, we believe a one-sided testing framework is appropriate. For this study we consider a one-sided test of the null hypothesis that the true primary outcome is no different between treatment and control with a type I error of alpha = 0.05. In order to have 80 % power to reject the null hypothesis if the alternative is true by using a one-sided Wilcoxon rank sum test at level alpha = 0.05 the required sample size for each treatment arm assuming equal allocation is 15. A maximum loss of 15 % is considered due to poor tissue quality or technical problems. Therefore a total of 36 patients will be enrolled and equally randomized to the two study arms.

### Data analysis

The frequency of CD8+ T cells (cells/mm^2^) in patients receiving 2 Gy preoperative radiotherapy versus no preoperative radiotherapy will be compared using the Wilcoxon rank sum test at a one-sided significant level alpha = 0.05.

Descriptive statistics will be used to summarize the distribution of CD8+ T cell counts by treatment arm (mean, median, range, inter-quartile-range, and standard deviation).

Descriptive statistics will be used to summarize the correlation of the two the methods (IHC and flow cytometry) of quantification of T cells. IHC will be used for primary analysis for which sample size estimation was performed. Distribution of T cell subsets (CD3+, CD4+, CD45RO+, Foxp3+) and other secondary endpoints of immune response (frequencies of tumour reactive T cells and protein levels) will be evaluated with same analysis plan. Correlation in quantitative measures of immune response will be evaluated using Spearman rank correlation.

Distribution of progression free survival (PFS) and overall survival (OS) will be estimated by the Kaplan-Meier method. The treatment groups will be compared with a stratified log rank test. The effect of frequencies of tumour infiltrating CD8+ T cells on PFS and OS will be analysed using Cox proportional hazards regression. The relationship between measures of immune response and PFS and OS will be explored in multivariable Cox proportional hazard models including treatment arm as covariate.

All secondary analyses will be exploratory using two-sided significance levels alpha = 0.05.

### Data management and quality assurance

The Department of Thoracic Surgery at Thoraxklinik and the Molecular and Radiation Oncology Department of the German Cancer Research Center and University Hospital Heidelberg coordinate this trial. Data regarding T cell detection in blood and bone marrow of all patients will be entered in a password-protected database at the Department of Thoracic Surgery at Thoraxklinik and at the German Cancer Research Center. The clinical and laboratory data of all included patients will be centrally collected in a password-protected database located at the Department of Thoracic Surgery at Thoraxklinik.

## Discussion

Non-small cell lung cancer (NSCLC) has still a poor prognosis. Even in patients with radically operated stage I NSCLC the 5-year recurrence-free rate only is about 75 % [8]. The development of additional treatments represents an unmet medical need for patients with completely resected NSCLC. Preclinical cellular immunotherapeutic approaches demonstrated the ability to reject solid tumours by tumour specific T lymphocytes [15, 48], the effectiveness of these approaches, however, could not be translated into clinical trials so far. Insufficient migration and activation of tumour infiltrating lymphocytes are considered the main reasons for inadequate host anti-tumour immune response. Lung cancer is supposed to exhibit a particular immunosuppressive microenvironment. Low dose ionizing radiation stimulates a localized pro-inflammatory milieu thereby modulating the tumour microenvironment. The results of this trial could demonstrate a beneficial immune related effect and suggest low dose radiation to be a complementary neoadjuvant component of immunotherapy prior to surgery in operable NSCLC with only mild side effects in future. However this needs to be investigated in larger trials. The goal of this randomized trial is to assess if combining preoperative low dose radiation and surgical removal of early stage non-small cell lung cancer will improve host anti-tumour immune response. If successful, a combination of immune modulatory treatments, such as ionizing radiation and checkpoint inhibition may help to reverse the process of immunosuppression in NSCLC and could therefore be an integral treatment strategy in NSCLC.
